# The importance of taking into account different perspectives of a health problem: the case of fibromyalgia

**DOI:** 10.1080/16549716.2017.1275189

**Published:** 2017-03-17

**Authors:** Anna-Britt Coe

**Affiliations:** ^a^ Epidemiology and Global Health Unit, Department of Public Health and Clinical Medicine, Umeå University, Umeå, Sweden

In 2014, Erica Briones defended her thesis ‘The Social Construction of Fibromyalgia as a Health Problem from the Perspective of Policies, Professionals and Patients [1]. I had the pleasure of being one of the examiners of her defense. Through her thesis, she makes several important contributions to knowledge about fibromyalgia. First, she uses an integral approach that incorporates three different perspectives of this phenomenon: those of policies, of providers, and of patients. Seldom do we find health research that takes us beyond one of these three perspectives, let alone attempts to integrate them and show how they dis/connect with one another. Briones does this in her cover story and five scientific articles.

Moreover, she examines how these perspectives of fibromyalgia are constructed across multiple social spheres: public and private, political and clinical, and work and domestic. In this way, her thesis helps break down what might be referred to as ‘tunnel vision’ or ‘separate silos’ in public health approaches that focus only on one of these spheres. Briones shows how policy, providers and patient perspectives are never limited to a single social sphere but encompass various spheres and these different spheres must take these into account to improve public health approaches.

In addition, Briones shows how gender inequality in society shapes the construction of these different perspectives and spheres. Rather than assuming that fibromyalgia results from a biomedical sex distinction between women and men, the thesis shows the impact of its gendered social construction making it into *a women’s disease*. This in turn makes fibromyalgia invisible in public policies, generates stigmatization in health care, and produces enormous difficulties among patients attempting to fulfill tasks and roles associated with their gender identity. It is these types of gender analysis, which draw upon gender theory in health and adopt a gender perspective throughout the study’s course, that are needed in public health research in order to not merely avoid furthering gender inequalities but contribute to eliminating these.

Finally, the thesis reflects systematic research whereby specific methods were selected to adapt to the different perspectives and spheres under inquiry, including a review of policies, in-depth interviews, and different analytical procedures. Clearly, to be able to effectively take into account and integrate different perspectives of a health problem, across different social spheres, and illuminate gender inequalities related to the problem, it is necessary to draw upon the range of methods available. As has been already shown in other fields, diversity is beneficial, and Briones shows this through her rich empirical material and analysis.

In sum, these contributions made by Briones through her thesis offer important insights for research, politics, and practices for addressing not only fibromyalgia but also other critical health concerns faced today.
